# A Lifestyle Intervention via Email in Minority Breast Cancer Survivors: Randomized Parallel-Group Feasibility Study

**DOI:** 10.2196/cancer.7495

**Published:** 2017-09-21

**Authors:** Raheem J Paxton, Richard Hajek, Patricia Newcomb, Megha Dobhal, Sujana Borra, Wendell C Taylor, Deborah Parra-Medina, Shine Chang, Kerry S Courneya, Gladys Block, Torin Block, Lovell A Jones

**Affiliations:** ^1^ Department of Community Medicine and Population Health The University of Alabama Tuscaloosa, AL United States; ^2^ Center for Health Equity and Evaluation Research The University of Texas MD Anderson Cancer Center Houston, TX United States; ^3^ Texas Health Resources Texas Health Harris Methodist Hospital Fort Worth, TX United States; ^4^ School of Public Health University of North Texas Health Science Center Fort Worth, TX United States; ^5^ Institute of Healthy Aging University of North Texas Health Science Center Forth Worth, TX United States; ^6^ Department of Health Promotion and Behavior Sciences The University of Texas Health Science Center at Houston Houston, TX United States; ^7^ Department of Mexican American & Latina/o Studies The University of Texas at Austin Austin, TX United States; ^8^ Department of Epidemiology The University of Texas MD Anderson Cancer Center Houston, TX United States; ^9^ Faculty of Physical Education and Recreation University of Alberta Edmonton, AB Canada; ^10^ Turnaround Health, a division of NutritionQuest Berkeley, CA United States; ^11^ School of Public Health University of California at Berkeley Berkeley, CA United States; ^12^ School of Public Health Texas A&M Health Science Center Bryan, TX United States

**Keywords:** breast neoplasm, African Americans, diet, feasibility study, physical activity, posture, program evaluation, Internet, computer tailoring, email

## Abstract

**Background:**

Our data have indicated that minority breast cancer survivors are receptive to participating in lifestyle interventions delivered via email or the Web, yet few Web-based studies exist in this population.

**Objective:**

The aim of this study was to examine the feasibility and preliminary results of an email-delivered diet and activity intervention program, “A Lifestyle Intervention Via Email (ALIVE),” delivered to a sample of racial and ethnic minority breast cancer survivors.

**Methods:**

Survivors (mean age: 52 years, 83% [59/71] African American) were recruited and randomized to receive either the ALIVE program’s 3-month physical activity track or its 3-month dietary track. The fully automated system provided tools for self-monitoring and goal setting, tailored content, and automated phone calls. Descriptive statistics and mixed-effects models were computed to examine the outcomes of the study.

**Results:**

Upon completion, 44 of 71 survivors completed the study. Our “intention-to-treat” analysis revealed that participants in the physical activity track made greater improvements in moderate to vigorous activity than those in the dietary track (+97 vs. +49 min/week, *P*<.001). Similarly, reductions in total sedentary time among those in the physical activity track (−304 vs. −59 min/week, *P*<.001) was nearly 5 times greater than that for participants in the dietary track. Our completers case analysis indicated that participants in the dietary track made improvements in the intake of fiber (+4.4 g/day), fruits and vegetables (+1.0 cup equivalents/day), and reductions in saturated fat (−2.3 g/day) and trans fat (−0.3 g/day) (all *P*<.05). However, these improvements in dietary intake were not significantly different from the changes observed by participants in the physical activity track (all *P*>.05). Process evaluation data indicated that most survivors would recommend ALIVE to other cancer survivors (97%), were satisfied with ALIVE (82%), and felt that ALIVE was effective (73%). However, survivors expressed concerns about the functionality of the interactive emails.

**Conclusions:**

ALIVE appears to be feasible for racial and ethnic minority cancer survivors and showed promising results for larger implementation. Although survivors favored the educational content, a mobile phone app and interactive emails that work on multiple email domains may help to boost adherence rates and to improve satisfaction with the Web-based platform.

**Trial Registration:**

ClinicalTrials.gov NCT02722850; https://clinicaltrials.gov/ct2/show/NCT02722850 (Archived by WebCite at http://www.webcitation.org/6tHN9VsPh)

## Introduction

Breast cancer survivors suffer from high rates of overweight or obesity and often do not meet current guidelines for physical activity and intake of fruits and vegetables [1-3]. Poor lifestyle habits of breast cancer survivors contribute to diminished health-related quality of life (HRQoL), increased risk of comorbid conditions, cancer recurrence, and premature mortality [2]. Unfortunately, even though minority breast cancer survivors suffer disproportionately from these circumstances, they remain underserved and underrepresented in epidemiological and intervention research [4-6]. Therefore, studies designed to improve the lifestyle behaviors of minority cancer survivors are warranted.

Comprehensive reviews and meta-analytic studies have indicated that clinic-based or in-person studies intended to improve diet, exercise, and HRQoL in cancer survivors have had promising results [2,7-10]. However, distance and time are fundamental barriers to participating in these studies [3,11]. Several researchers have advocated for home-based interventions that include telephone counseling or tailored print materials [12,13]. Whereas many home-based programs have led to significant improvements in healthy lifestyle behaviors [14-20], they are not always sustainable because telephone counseling and mass mailings require significant personnel effort. Studies that utilize the Web offer a potential to overcome the challenges (cost, time, and distance) experienced in traditional home-based interventions [21]. Given these benefits, there has been an increase in advocacy for Web-based interventions [22,23], especially those designed for cancer survivors [24-31]. Previous Web-based studies developed for cancer survivors have observed significant improvements in lifestyle behaviors [28,29,31-34].

Despite the recent surge in Web-based interventions among cancer survivors, few studies have focused on minority cancer survivors [34]. Also, the majority of the studies have focused primarily on physical activity [25-28,30,31], with only a few intervening on dietary intake [24,29,32]. Therefore, we proposed to address this limitation by testing the feasibility and preliminary effects of a previously developed fully automated system that utilizes weekly emails, self-monitoring and goal-setting tools, and automated counseling phone calls to improve physical activity and dietary intake [35]. We utilized an evidence-based program entitled “A Lifestyle Intervention Via Email” (ALIVE) [36]. In previous research, ALIVE demonstrated improvements in moderate to vigorous physical activity and fruit and vegetable consumption as well as reductions in saturated and trans fat in a sample of healthy worksite employees. In this study, participants were randomized to either a physical activity or a dietary track. We hypothesized that survivors randomized to the physical activity track would experience greater improvements in moderate and vigorous physical activity than those randomized to the dietary track. Similarly, we hypothesized that survivors randomized to the dietary track would experience greater improvements in fruit and vegetable consumption and reductions in saturated and trans fats than those randomized to the physical activity track.

## Methods

### Recruitment and Consent

Minority cancer survivors were recruited using nonprobability sampling techniques. Survivors were identified via word of mouth, existing relationships with community-based organizations, and cases ascertained from tumor registries in the North Texas metropolitan area. Eligibility criteria included (1) a previous diagnosis of breast cancer, (2) being at least 18 years old at study enrollment, (3) having completed treatment (except hormonal therapy) at least 6 months before study enrollment, and (4) receptivity to participating in a Web-based intervention study. Also, those who self-identified as African American, Hispanic, or of mixed ethnicity (ie, Asian and African American or African American and non-Hispanic white) were eligible for this study. We used a rolling recruitment process for screening and consenting participants. Survivors completed the screening and consent process from June 2014 to October 2015 using a multi-gated approach. All identified survivors were screened with Web-based surveys that assessed prior history of cancer, lifestyle factors (ie, diet and exercise), and physical activity readiness. The Physical Activity Readiness Questionnaire (PAR-Q) was used to identify contraindications to physical activity[37]. In the event where contraindications were identified, participants were asked to provide information indicating physician approval. Survivors with invalid data or who were not identified as cancer survivors were ineligible. Once survivors completed the screening survey, they were directed to a separate link containing a Web-based consent form. The screening and consent links were distinct from those later delivered for the ALIVE website. Ethical approval by the University of North Texas Health Science Center and participating health care institutions was established before enrolling survivors (Clinical trial registration number, NCT02722850).

### Randomization and Enrollment

After participants completed the screening and consent process, a random number generator was used to randomize survivors to either a 3-month physical activity or a 3-month dietary track. Survivors were then sent track-specific enrollment links (ie, physical activity or dietary intake) to begin the ALIVE intervention. Participants in the dietary track could further choose between changing their dietary fat and added sugar intake or their fruit and vegetable intake. Data from participants working on both dietary behaviors were treated as one diet track for this analysis. A total of 71 minority survivors were randomized with equal probability to each track. Survivors received a US $20 incentive for completing each assessment. Thus, if they completed the baseline and follow-up assessment, they received a total of US $40.

### Study Goals

Survivors in the physical activity track were encouraged to meet or exceed current federal recommendations for physical activity (eg, ≥150 min of moderate to vigorous physical activity per week). Survivors in the fruit and vegetable subtrack were encouraged to meet or exceed current recommendations for fruit and vegetable intake (eg, ≥3.5 cup servings of fruit and vegetable consumption). Survivors in the fats and added sugar track were encouraged to decrease intake of saturated and trans fats, decrease added sugars, and increase the intake of “good” fats and carbohydrates to meet or exceed these health recommendations (ie, ≤50 g/day of added sugars and ≤10% of calories from saturated fat) [38]. Content and messages provided to survivors were track specific and designed to promote a target behavior or behaviors.

### Intervention

ALIVE was developed in collaboration between the Kaiser Permanente of Northern California Division of Research and NutritionQuest. No tailoring or modifications were made to the original program for this study. ALIVE was a theory-based coaching system derived from the principles of various theoretical models including the social cognitive theory [39,40], goal-setting theory [41], social marketing [42], and the transtheoretical model [43]. It was designed to enable participants to break up large goals into small achievable goals that could be accomplished weekly. ALIVE was delivered to survivors via an individualized website and interactive emails delivered weekly. At baseline, survivors were asked to complete a diet and activity health risk assessment. The risk assessment provided tailored feedback based on assessed levels of diet and activity and a planning tool to guide improvements in track-specific behaviors. Behavior change strategies such as goal setting, self-monitoring, rewards, cues to action, and repetition were incorporated throughout the program. Functions and features of the ALIVE program were identical across tracks, whereas content (eg, recommended goals and health education materials) differed by track. ALIVE uses participant-reported diet and activity behaviors to individualize the weekly goals it recommended to participants. A brief description of the ALIVE components are reported in Table 1.

### Measures

#### Physical Activity and Sedentary Behavior

The Physical Activity Questionnaire (PAQ) was adapted from the Cross-Cultural Activity Participation Study (CAPS) Questionnaire [44]. It comprised 34 domain-specific activities (ie, household and caregiving, sedentary, transportation-related activities, and leisure and sport-related activities). Survivors were asked to indicate how many days per week and minutes per day they participated in each of the activities in a typical week. For the purpose of this study, minutes of moderate to vigorous physical activity per week were utilized as our physical activity outcome. In addition, estimates were derived from several forms of sedentary behavior (ie, total, discretionary, television-viewing, and other), which served as a separate outcome. Test-retest reliability of the instrument utilized in the original ALIVE study indicated adequate reliability [35]. Physical activity and sedentary behaviors were assessed at the baseline and 3-month assessment via the ALIVE system.

#### Dietary Intake

The dietary questionnaire queried participants on the intake of 35 commonly consumed foods identified as significant contributors to the intake of fruits and vegetables, added sugars, and saturated and trans fats in the National Health and Nutrition Examination Survey [45]. Survivors were asked to report the frequency and portion size of each of the 35 items and the subtype of select items (eg, diet soda vs non diet soda). The items included commonly consumed foods (eg, hamburgers), fruits and vegetables, nuts, grains (eg, cereals), processed meats (eg, hot dogs), sweets (eg, candy, pastries, and cookies), dairy (eg, milk, eggs, and cheese), and juices (eg, 100% fruit juice and Hi-C). The response scale ranged from items they consumed multiple times daily to items they consumed only a few times per month. Nutrient estimates were calculated based on consumption patterns and usual portion sizes consumed. The resulting nutrient estimates were derived from established databases [46,47]. The dietary items had acceptable test-retest reliability in the original ALIVE study [35]. Dietary intake was assessed at the baseline and 3-month assessment via the ALIVE system.

#### Process Evaluation and Feasibility

Survivors were asked to report on their satisfaction with components of the ALIVE system in a separate Web-based survey. Satisfaction was rated on a 5-point Likert-type response scale ranging from 1 (very dissatisfied) to 5 (very satisfied). We also included a separate overall satisfaction question. We used one question to assess the perceived effectiveness of ALIVE to change health behaviors and another question to assess whether they would recommend ALIVE to other cancer survivors (yes or no). Finally, we included open-ended questions that provided survivors with the opportunity to report on three likes and three dislikes about the ALIVE program. Our process evaluation facilitated our ability to assess the following components of feasibility: acceptability (ie, satisfaction), demand (ie, adherence to website usage), implementation and practicality (ie, success or failure of execution reported in the qualitative responses), and limited efficacy (ie, change scores and effect sizes) [48].

#### Sociodemographic and Medical Data

These self-report data were collected during the screening survey. The data included items related to age, education, employment status, age at diagnosis, disease stage at diagnosis, and comorbid conditions. We summed the number of comorbid conditions (ie, arthritis, diabetes, high blood pressure, heart disease, and high cholesterol) to create a single continuous variable.

**Table 1:** Components of the ALIVE (A Lifestyle Intervention Via Email) program by study track.

Features	Physical activity	Dietary intake
Individual tailoring: Information used to tailor content was based on the baseline diet and physical activity survey.	Preference for facility-based or home-based exercises Stage of physical activity readiness Social support for exercise Physical activity barriers Suggestions to reduce sedentary behavior User home page	Habits related to cooking and eating out Stage of dietary readiness Specific foods consumed Social support for healthy eating Dietary barriers Suggestions to reduce the top three sources of problematic nutrients User home page
Tailored goal setting: Content encouraging progress toward goal attainment. New goals were set once old ones were accomplished.	Weekly emails suggesting four to six small-step goals tailored to characteristics mentioned above (eg, I will walk 5 min at lunch time today) Queries about physical activity goal achievement	Weekly emails suggesting four to six small-step goals tailored to characteristics mentioned above (eg, I will have a salad at lunch one day this week) Queries about dietary goal achievement
Midweek reminders	Reminded survivors of their physical activity goals	Reminded participants of their dietary goals
Tips: Tips sent out weekly.	Tips provided information related to achieving physical activity goals and overcoming specific physical activity barriers	Tips provided information related to achieving dietary goals and overcoming specific dietary barriers
Goal tracker: Tracks which goals survivors achieve.	Tracked goals related to the frequency, type, and duration of physical activity	Tracked goals related to the frequency, type, and quantity of dietary nutrients
Simulation tool: An interactive feature of the ALIVE website for simulating effects of recommended goals	Allowed the participant to simulate how changing the frequency, quantity, or type of specific activities impacts total physical activity levels	Allowed the participant to simulate how changing the frequency, quantity, or type of specific foods or beverages impacts total nutrient levels
Health notes: Each week, a different topic was discussed.	Topics included research on the relationship between physical activity and various health outcomes	Topics included research on the relationship between a healthy diet and various health outcomes
Provisions for social support: Weekly goals and tips encouraging survivors to build a support systems with friends and family members. Chat rooms were available for participants to discuss problems and offer solutions to each other.	Provided suggestions such as walks with colleagues at lunch time Allowed survivors to engage and troubleshoot physical activity barriers and solutions.	Provided suggestions to eat healthy meals with friends and family Allowed survivors to engage and troubleshoot dietary barriers and solutions.
Automated phone calls: 3- to 5-min calls that facilitated goal setting, provided positive words of encouragement, and emphasized stage specific processes of change. Survivors also queried about personal barriers and goals.	Calls encouraged: Scheduling physical activity Overcoming physical activity barriers Making public commitments to be active Identifying a workout partner Reporting your physical activity achievements to others Encouraging friends to hold you accountable to activity goals	Calls encouraged: Planning healthy meals Overcoming dietary barriers Making public commitments to consume a healthy diet Identifying a friend who would go out and consume a healthy meal with you Reporting your dietary achievements to others Encouraging friends to hold you accountable to your dietary goals

### Statistical Analysis

Descriptive statistics were computed to describe the study population. Chi-square tests for independence and Fisher exact tests were used to determine whether there were categorical differences in the sociodemographic and medical variables between study tracks. Subsequent nonparametric Wilcoxon rank-sum tests were computed to determine whether there were mean or median differences in the continuous outcomes at baseline. Generalized mixed-effects models (PROC GLIMMIX) were used to estimate within and between-group changes in study outcomes over time. Given that many of the outcomes were nonnormal, log-normal or Poisson distributions were specified. The effects in the model comprised time, track, time by track interaction, and significant covariates identified in the initial analyses. Furthermore, survivors nested within study tracks were treated as a random effect. Cohen *d* values were also computed to estimate the effect size. Separate analyses were conducted for cases with complete data and for those where an intention-to-treat (ITT) protocol was applied. To account for missing data in our intention-to-treat analysis, the last observation was carried forward. Furthermore, descriptive statistics were computed for process evaluation data, and *t* tests were used to make comparisons between the two study tracks. All data were analyzed using Statistical Analysis System (SAS) version 9.3 (SAS Institute Inc, Cary NC), and statistical significance was determined a *P* value of ≤.05 with a two-sided test.

## Results

### Descriptive Statistics

#### Recruitment and Consent

**Figure 1:**
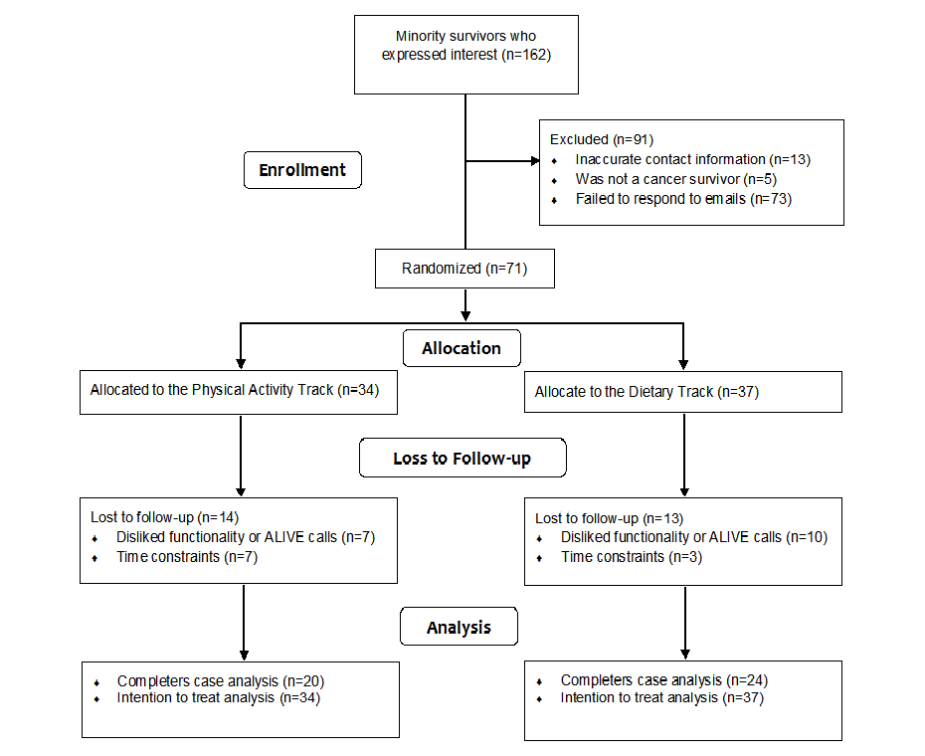
Consolidated standards of reporting trials (CONSORT) diagram.

In total, 162 minority survivors expressed interest in participating in the study, but only 71 of them (43.8%, 71/162) received the allocated intervention materials (see Figure 1). Unfortunately, 86 of the 162 persons who expressed interest in the study provided incorrect email addresses (N=13) or failed to return follow-up emails and phone calls (N=73). The randomized survivors were on average 52 years old at study enrollment, which was 8 years after initial cancer diagnosis. Most were African American (83%, 59/71), college educated (65%, 46/71), and diagnosed with regional stage disease (54%, 38/71). Most failed to meet guidelines for intake of fruit and vegetables (72%, 51/71) and saturated fat (61%, 43/71). Roughly, half were obese (52%, 37/71), whereas a surprising number (63%, 45/71) were already meeting current guidelines for physical activity at baseline (these data are not shown).

**Table 2:** Descriptive statistics comparing completers and noncompleters at baseline.

Variables		Total sample N=71	Completers n=44	Noncompleters n=27	*P* value^a^
**Mean age (SD**^b^**)**		52.2 (8.6)	52.0 (7.8)	52.6 (9.9)	.62
	Median and range of age	53 (26-72)	52 (32-69)	54 (26-72)	-
Mean age at diagnosis (SD)		43.9 (8.9)	43.3 (7.2)	44.8 (11.2)	.21
Mean years out from diagnosis (SD)		8.4 (6.5)	8.8 (6.9)	7.7 (5.8)	.57
**Race or ethnicity, n (%)**					.86
	African American	59 (83)	36 (61)	23 (39)	
	Hispanic	8 (11)	5 (63)	3 (37)	
	Mixed	4 (6)	3 (75)	1 (25)	
Currently employed, n (%)		51 (72)	33 (75)	18 (67)	.45
**Education, n (%)**					.20
	College graduate	46 (65)	31 (70)	15 (56)	
**Stage, n (%)**					.60
	Localized	14 (20)	9 (21)	5 (19)	
	Regional	38 (54)	23 (52)	15 (56)	
	Distant	19 (26)	12 (27)	7 (25)	
**Treatment, n (%)**					
	Surgery	67 (94)	41 (93)	26 (96)	.37
	Radiation	49 (69)	30 (68)	19 (70)	.85
	Chemotherapy	53 (75)	33 (75)	20 (74)	.93
	Hormone	31 (44)	19 (43)	12 (44)	.92
**Number of comorbidities, mean (SD)**		0.8 (0.9)	0.8 (1.1)	0.7 (0.7)	.93
	Median and range of comorbidities	1 (0-4)	1 (0-4)	1 (0-2)	-
**Select lifestyle behaviors, mean (SD)**					
	Body mass index	30.8 (6.0)	30.5 (5.8)	31.3 (6.6)	.66
	Fruit and vegetable intake in cup servings	2.8 (1.6)	2.7 (1.6)	3.0 (1.6)	.48
	Fiber intake in g/day	16.4 (8.1)	16.2 (7.9)	16.7 (8.6)	.84
	Saturated fat in % calories	11.8 (7.7)	11.8 (7.7)	11.7 (7.9)	.84
	Minutes of moderate to vigorous physical activity/week	222 (272)	240 (233)	194 (329)	.19
	Total sedentary minutes/week	1462 (886)	1412 (853)	1554 (949)	.65

^a^Categorical *P* values are based on chi-square or Fisher exact test, whereas continuous *P* values are based on nonparametric Wilcoxon rank-sum test.

^b^SD: standard deviation.

Attrition at the 3-month assessment was 38% (27/71), with no differences in attrition observed between completers and noncompleters on lifestyle, treatment-related variables, and sociodemographic characteristics (all *P*>.05). Descriptive statistics comparing completers and noncompleters are described in Table 2.

#### Baseline Differences Between Study Tracks

At the baseline assessment, Hispanic survivors were more likely to be randomized to the physical activity track, and mixed race individuals were more likely to be randomized to the dietary track (*P*=.02). Descriptive statistics comparing survivors in the diet and physical activity tracks are reported in Table 3.

**Table 3:** Descriptive statistics of participants enrolled in ALIVE (A Lifestyle Intervention Via Email) by study tracks at baseline.

Variables		Physical activity N=34	Diet N=37	*P* value^a^
Dropout, n (%)		14 (41)	13 (35)	.60
Mean age (SD^b^)		52.7 (8.4)	51.8 (8.9)	.70
Mean age at diagnosis (SD)		44.6 (7.8)	43.3 (9.9)	.52
Mean years out from diagnosis (SD)		8.2 (5.6)	8.5 (7.1)	.96
**Race or ethnicity, n (%)**				.02
	African American	27 (79)	32 (86)	
	Hispanic	7 (21)	1 (3)	
	Mixed or other	0 (0)	4 (11)	
Employment, n (%)		26 (76)	25 (68)	.41
**Education, n (%)**				
	College graduate	25 (74)	21 (57)	.14
Number of comorbidities, mean (SD)		0.8 (0.8)	0.8 (1.1)	.57
**Stage, n (%)**				.16
	Localized	10 (29)	4 (11)	
	Regional	16 (47)	22 (59)	
	Distant	8 (24)	11 (30)	
**Treatment, n (%)**				
	Surgery	31 (91)	36 (97)	.34
	Radiation	23 (68)	26 (70)	.81
	Chemotherapy	22 (65)	31 (84)	.07
	Hormone	15 (44)	16 (43)	.94
**Lifestyle behaviors, median (25%-75%)**^c^				
	Body mass index	29.8 (25.8-34.1)	31.0 (25.8-35.8)	.50
	Fruit and vegetable intake in cup servings	2.5 (1.4-4.1)	2.8 (1.5-3.6)	.80
	Fiber intake in g/day	15.8 (10.7-19.7)	15.4 (10.2-21.6)	.86
	Sugar in g/day	14.8 (7.2-44.5)	24.5 (14.1-51.3)	.19
	Carbohydrates in g/day	113.7 (84.8-197.5)	142.2 (106.6-186.0)	.28
	Trans fat in % calories	0.4 (0.2-0.8)	0.5 (0.3-0.9)	.21
	Saturated fat in % calories	8.8 (5.6-13.4)	11.2 (6.7-15.1)	.14
	Minutes of moderate to vigorous physical activity/week	138 (0-390)	150 (0-390)	>.99
	Discretionary minutes of sedentary time/week	1095 (660-1680)	1170 (510-1860)	.93
	Other minutes of sedentary time/week	210 (150-720)	360 (120-720)	.70
	Television viewing time/week	840 (420-1260)	720 (360-1200)	.62
	Total sedentary minutes/week	1410 (750-2040)	1380 (630-1890)	.53

^a^Categorical *P* values are based on chi-square or Fisher exact test, whereas continuous *P* values are based on nonparametric Wilcoxon rank-sum test.

^b^SD: standard deviation.

^c^The median and 25% and 75% CIs were reported for the lifestyle variables.

### Intervention Outcomes

#### Physical Activity

Our “completers only” and ITT analyses are reported in Tables 4 and 5, respectively. Both tracks made improvements in physical activity (all *P*<.001), but the improvements in the physical activity track were greater than that of the dietary track (all *P*<.001). In particular, the improvements in minutes of moderate physical activity per week were more than twice than that of the dietary track in the completers (+165 vs +75 min/week; *P*<.001) analysis and nearly two times greater in the ITT (+97 vs +49 min/week; *P*<.001) analysis.

#### Sedentary Behavior

Our analyses indicated that both groups made reductions in discretionary, television-related, and total sedentary time (all *P*<.001), but the reductions in the physical activity track were greater than that of the dietary track (all *P*<.001). In particular, the reductions in discretionary and television-related sedentary time were more than double than that of the dietary track in both the completers and ITT analyses. More importantly, the reduction in total sedentary time observed among the physical activity track was more than five times (−517 vs −91 min/week; *P*<.001) than that of the dietary track in the completers analysis and nearly five times (−304 vs −59 min/week; *P*<.001) than that of the dietary track in the ITT analysis.

#### Dietary Intake

Our completers case analysis indicates that only the dietary track made improvements in the intake of fiber (+4.4 g/day; *P*=.01), fruits and vegetables (+1.0 cup servings/day; *P*=.002), saturated fat (−2.8 g/day; *P*=.03), and trans fat (−0.3 g/day; *P*=.04). In the ITT analysis, only fruit and vegetable intake (+0.7 cup servings/day; *P*=.002) improved in the dietary track. The changes observed in our dietary track did not differ from the changes observed in the physical activity track in both the completers case and ITT (all *P*>.05) analyses.

**Table 4:** Change scores for the study outcomes in the completers case analysis (N=44).

Outcomes	Physical activity change^a^ (SE^b^) N=20	Dietary intake change^a^ (SE) N=24	Effect size	*P* value^c^
Minutes of moderate to vigorous physical activity/week	+165 (68)^d^	+75 (62)^d^	0.30	<.001
Discretionary minutes of sedentary time/week	−309 (138)^d^	−125 (126)^d^	0.30	<.001
Other minutes of sedentary time/week	−93 (75)^d^	+23 (68)^d^	0.35	<.001
Television viewing time/week	−216 (114)^d^	−103 (104)^d^	0.22	<.001
Total sedentary minutes/week	−517 (148)^d^	−91 (135)^d^	0.64	<.001
Sugar in g/day	+6.6 (4.4)	−2.3 (4.0)	0.45	.43
Fiber in g/day	+1.9 (1.7)	+4.4 (1.6)^e^	0.32	.40
Fruits and vegetables in cup equivalents/day	+0.6 (0.3)	+1.0 (0.3)^d^	0.28	.35
Saturated fat in g/day	−1.0 (1.3)	−0.8 (1.2)^e^	0.31	.46
Trans fat in g/day	+0.0 (0.2)	−0.3 (0.1)^e^	0.51	.99
Carbohydrates in g/day	+14.2 (11.3)	+17.6 (10.3)	0.07	.68

^a^All values represent within-group mean changes for the variables between baseline and follow-up periods.

^b^SE: standard error.

^c^Mixed-effects models were adjusted for race or ethnicity.

^d^*P*<.01.

^e^*P*<.05.

**Table 5:** Change scores for the study outcomes in the intention-to-treat analysis (N=71). An intention-to-treat protocol was applied where the last observations were carried forward.

Outcomes	Physical activity change^a^ (SE^b^) N=34	Dietary intake change^a^ (SE) N=37	Effect size	*P* value^c^
Minutes of moderate to vigorous physical activity/week	+97 (42)^d^	+49 (40)^d^	0.20	<.001
Discretionary minutes of sedentary time/week	−182 (85)^d^	−81 (81)^d^	0.20	<.001
Other minutes of sedentary time/week	−55 (45)^d^	−15 (43)^e^	0.15	<.001
Television viewing time/week	−127 (69)^d^	−66 (67)^d^	0.15	<.001
Total sedentary minutes/week	−304 (94)^d^	−59 (90)^d^	0.45	<.001
Sugar in g/day	+3.9 (2.7)	−1.5 (2.5)	0.35	.42
Fiber in g/day	+1.1 (1.1)	+2.9 (1.1)	0.27	.35
Fruits and vegetables in cup equivalents/day	+0.3 (0.2)	+0.7 (0.2)^e^	0.34	.29
Saturated fat in g/day	−0.6 (0.8)	−1.8 (0.8)	0.25	.40
Trans fat in g/day	−0.0 (0.1)	−0.2 (0.1)	0.30	.90
Carbohydrates in g/day	+8.3 (6.9)	+11.4 (6.6)	0.08	.61

^a^All values represent within-group mean changes for the variables between baseline and follow-up periods.

^b^SE: standard error.

^c^Mixed-effects models were adjusted for race or ethnicity.

^d^*P*<.01.

^e^*P*<.05.

### Process Evaluation and Feasibility

#### Demand

Website usage did not differ between study intervention conditions. Survivors in the physical activity track visited the website on an average of 9.6 of the 12 weeks, whereas survivors in the diet track visited the website on an average of 10.7 of the 12 weeks (*P*=.15).

#### Satisfaction

Survivors in both tracks were mostly satisfied with the following components: tips for overcoming barriers, tips for achieving goals, goal-setting tools, and health notes. Additionally, most (97%) who completed the follow-up assessment indicated that they would recommend the ALIVE program to other cancer survivors. No statistically significant differences were observed between tracks. However, mean scores for the tracking tools were marginally lower in the physical activity track (*P*=.05). Mean satisfaction scores by track are reported in Table 6.

#### Implementation and Practicality

This component of feasibility was assessed via the qualitative responses obtained during our process evaluation. “Likes” reported by survivors could be grouped into six main themes: educational information (36%), email reminders (14%), goal-setting tools (12%), ease of use (9%), and motivation or encouragement (9%). The most commonly reported theme related to the educational information presented by the ALIVE program. For example, survivors indicated they liked the “information and tips,” and the “Did you know section.”

Components of ALIVE that survivors did not like could be grouped into the following themes: Functionality (48%), information (31%), tools (14%), and time (7%). For functionality, survivors indicated that they “could not enter goals,” that “links were not supported” or that they “got stuck” at some point while using the website. Examples of comments pertaining to information were “too much information” and “no relevant patient information.”

#### Limited Efficacy

The effect sizes measuring changes in dietary intake between tracks were mostly medium in size. In the completers case analysis (see Table 4), effect sizes ranged from 0.28 for fruit and vegetable intake to 0.45 for sugar intake. In the ITT analysis (see Table 5), effect sizes were more modest but similar in magnitude (range=0.25 for saturated fat intake to 0.35 for added sugar intake). The effect sizes measuring changes in physical activity and sedentary behavior between tracks differed by the variable of interest and analysis. In both the completers case and ITT analysis, the effect sizes were small for television viewing (0.22 for completers case and 0.15 for ITT analysis). However, for total sedentary time, the effect sizes were mostly large (0.64 for completers case and 0.45 for ITT analysis). For physical activity, the effect sizes were small (0.20) for the ITT analysis but medium for the completers case analysis (0.30).

**Table 6:** Process evaluation data for study participants.

Satisfaction (1=not at all, 5=very satisfied)	Total	Physical activity	Diet	*P* value^a^
Tips for overcoming barriers	4.2 (0.6)	4.1 (0.7)	4.2 (0.6)	.63
Tips for achieving goals	4.2 (0.6)	4.2 (0.7)	4.3 (0.6)	.78
Tracker of daily habits	3.7 (0.8)	3.4 (0.8)	4.0 (0.8)	.05
Progress tools—tracks current and past goals	3.9 (0.9)	3.6 (1.0)	4.2 (0.7)	.08
Simulator tools—tool to help you visualize success	4.0 (0.7)	4.0 (0.7)	4.0 (0.6)	.99
Goal-setting tools	4.2 (0.7)	4.3 (0.7)	4.1 (0.8)	.46
Automated phone coaching	3.5 (1.3)	3.4 (1.2)	3.6 (1.3)	.68
Tailored newsletters	4.0 (0.9)	4.1 (0.8)	3.9 (1.0)	.57
Health note—articles to increase knowledge and skills	4.2 (0.9)	4.2 (0.8)	4.1 (1.0)	.85
Overall satisfaction	4.1 (0.9)	3.9 (1.0)	4.3 (0.7)	.24
Effectiveness in changing behavior (1=not at all, 5=very effective)	3.8 (0.9)	3.7 (1.1)	3.8 (0.7)	.67
Recommend ALIVE^b^ to other survivors, % yes	97	95	100	.47

^a^*t* tests were used to compare continuous indicators, and chi-square test of independence were used to compare the single binary item.

^b^ALIVE: A Lifestyle Intervention Via Email.

## Discussion

### Principal Findings

In this randomized parallel-group study, we observed that survivors randomized to the physical activity track made greater improvements in physical activity and greater reductions in sedentary behavior than those randomized to the dietary track. Despite the improvements in our activity-related constructs, these data only partially support our initial hypotheses, given that changes in the dietary variables did not differ significantly between tracks. Our process evaluation indicated that survivors were mostly satisfied with ALIVE and would recommend it to other survivors. However, concerns about ALIVE were noted. Overall, these data demonstrate that Web-based interventions such as ALIVE are feasible for racial and ethnic minority breast cancer survivors, but challenges must be addressed to improve the end user experience. The Alive program developers have recently developed and tested an updated version of the program that addresses some of the concerns identified in this study.

This is one of the first studies to examine the feasibility of a fully automated Web-based intervention in a sample of underserved breast cancer survivors. Our feasibility data were favorable, but attrition rates were high. The study’s attrition rate was comparable to previous Web-based intervention studies [49-51] but higher than recent studies conducted among cancer survivors [24,26,29-31,34]. Our team discovered that functionality challenges contributed to high attrition rates. Challenges reported by survivors included repeat calls from the automated phone system and ALIVE email messages not being fully interactive within certain email domains (ie, AOL, Thunderbird, Live, Outlook, and Lotus) nor on mobile phones or tablets. Therefore, many survivors were only able to access ALIVE from a desktop computer. The challenges resulted in considerable frustration and many asked to be removed from the study. Unfortunately, our team was not aware of the technical difficulties before the study. However, we worked with NutritionQuest to address the challenges and identify solutions for participants. Encouragingly, our process evaluation was overwhelmingly positive, despite the challenges.

ALIVE was associated with significant improvements in physical activity for both tracks. Prior Web-based interventions among cancer survivors have observed significant improvements in physical activity [24,28-31,33,34], which ranged from 18 min [24] to 103 min [30]. Importantly, in our physical activity track, we observed a 165-min increase in our completers analysis and a 97-min increase in our ITT analysis. Despite these broad improvements, our effect sizes were small to medium in magnitude. The small effect sizes may be due to transfer effects [52], whereby setting goals in one’s behavior increases one’s confidence, intentions, and motivation to make improvements in another behavior [53-55]. Here, setting goals for diet may have transferred over to physical activity. Transfer effects may be common among cancer survivors because they capitalize on the “teachable moment” following their cancer diagnosis.

To our knowledge, this is one of the first Web-based studies among cancer survivors to observe significant changes in sedentary time. ALIVE was not designed to be a sedentary behavior intervention, yet reductions in sedentary time were observed among our physical activity track. In discussions with NutritionQuest to inquire about the sedentary behavior curriculum, we were informed that educational materials discussing sedentary behaviors were minimal. Observed improvements in sedentary activity could be the result of this minimal sedentary behavior program content. Alternatively, it could be a transfer effect, similar to what was observed in dietary track. More research is needed to determine how, when, and for whom transfer effects occur.

Few Web-based interventions for cancer survivors have intervened on dietary intake. Our completers case analysis indicated significant improvements in the intake of fiber, fruits and vegetables intake, and saturated fat for the dietary track. These data support the results found in the original ALIVE study [36]. However, the observed changes did not differ significantly between tracks. Additionally, similar results were not observed in our ITT analysis. To our knowledge, only three Web-based intervention studies among cancer survivors have intervened on dietary intake [24,29,32], with two studies showing improvements [29,33]. It could be that 3 months were not sufficient to produce changes in dietary intake in our sample. Recently, Kanera et al [33] demonstrated an improvement in dietary intake at 12 months; a diet effect was not significant at the 6-month assessment [32]. More research is needed to determine the recommended program length required to change behavioral outcomes in Web-based intervention studies.

### Limitations

Our study has limitations. Our team used a convenient sampling strategy to maximize our recruitment efforts, and our sample consisted mostly of African American survivors who were college educated. It should also be noted that eligibility was not based on baseline physical activity or dietary behaviors. In particular, some participants were meeting guidelines for physical activity or dietary intake before joining the study. This may have lowered our estimated effect size between study tracks. Prior studies have observed stronger effects among survivors not meeting guidelines to lifestyle behaviors at the baseline assessment [50]. Furthermore, our attrition rate was high, and many survivors did not return our emails or calls lowering our accrual rate. We are uncertain why participants never returned our emails or calls. Our team can only speculate that our emails with embedded links to the ALIVE websites were identified as junk mail and never received by the survivors. Other survivors who failed to complete the study were either not sufficiently engaged, were frustrated by technical challenges, or had competing priorities that reduced their interest in completing the study. Finally, our outcome measures were self-report and subject to recall and reporting biases. Self-report surveys are common in Web-based interventions, where obtaining objective estimates of physical activity and dietary intake would be costly. Despite the limitations, there are several strengths, including (1) a focus on high-priority breast cancer survivors, (2) significant or positive trends in lifestyle behavior changes, and (3) use of an evidence-based intervention tool with demonstrated efficacy in healthy adults.

### Conclusions

ALIVE appears to be feasible for racial and ethnic minority breast cancer survivors and capable of improving multiple lifestyle behaviors. Although we observed favorable ratings for ALIVE, improvements to functionality and a tailoring to cancer survivors are warranted. Web-based programs should be created to minimize challenges that the end user would encounter. Since the time our study concluded, the developers of ALIVE have released an updated version of the program that includes features to increase engagement and reduce attrition. In particular, the newest version of ALIVE was designed to operate on phones, tablets, and computers; includes a stand-alone mobile phone app; and uses gamification, a points system, and other strategies to increase adherence [56]. Additional studies are needed to test the platform utilized here as well as the newest version of ALIVE in a sample of breast cancer survivors. Such studies could recruit a larger and more ethnically diverse sample to explore similarities and differences in the adoption and maintenance of lifestyle behaviors. Fully automated programs such as ALIVE are capable of being incorporated with minimal cost in clinical and community-based settings with the potential for dissemination and implementation to thousands of cancer survivors nationwide.

## Appendix

Multimedia Appendix 1 CONSORT-EHEALTH checklist (v1.6.1).

## Abbreviations

ALIVE: A Lifestyle Intervention Via Email
CAPS: Cross-Cultural Activity Participation Study
HRQoL: health-related quality of life
ITT: intention-to-treat
SAS: Statistical Analysis System
SE: standard error
SD: standard deviation
PAR-Q: Physical Activity Readiness Qestionnaire
PAQ: Physical Activity Questionnaire

## References

[1] Blanchard CM, Courneya KS, Stein K, American Cancer Society's SCS-II (2008). Cancer survivors' adherence to lifestyle behavior recommendations and associations with health-related quality of life: results from the American Cancer Society's SCS-II. J Clin Oncol.

[2] Rock CL, Doyle C, Demark-Wahnefried W, Meyerhardt J, Courneya KS, Schwartz AL, Bandera EV, Hamilton KK, Grant B, McCullough M, Byers T, Gansler T (2012). Nutrition and physical activity guidelines for cancer survivors. CA Cancer J Clin.

[3] Demark-Wahnefried W, Peterson B, McBride C, Lipkus I, Clipp E (2000). Current health behaviors and readiness to pursue life-style changes among men and women diagnosed with early stage prostate and breast carcinomas. Cancer.

[4] Adams SA, Butler WM, Fulton J, Heiney SP, Williams EM, Delage AF, Khang L, Hebert JR (2012). Racial disparities in breast cancer mortality in a multiethnic cohort in the Southeast. Cancer.

[5] Leopold C, Wagner AK, Zhang F, Lu CY, Earle C, Nekhlyudov L, Degnan D, Frank WJ (2016). Racial disparities in all-cause mortality among younger commercially insured women with incident metastatic breast cancer. Breast Cancer Res Treat.

[6] Samson ME, Adams SA, Orekoya O, Hebert JR (2016). Understanding the association of type 2 diabetes mellitus in breast cancer among African American and European American populations in South Carolina. J Racial Ethn Health Disparities.

[7] Schmitz KH, Holtzman J, Courneya KS, Mâsse LC, Duval S, Kane R (2005). Controlled physical activity trials in cancer survivors: a systematic review and meta-analysis. Cancer Epidemiol Biomarkers Prev.

[8] Speck RM, Courneya KS, Mâsse LC, Duval S, Schmitz KH (2010). An update of controlled physical activity trials in cancer survivors: a systematic review and meta-analysis. J Cancer Surviv.

[9] Focht BC, Clinton SK, Devor ST, Garver MJ, Lucas AR, Thomas-Ahner JM, Grainger E (2013). Resistance exercise interventions during and following cancer treatment: a systematic review. J Support Oncol.

[10] Rock CL, Demark-Wahnefried W (2002). Nutrition and survival after the diagnosis of breast cancer: a review of the evidence. J Clin Oncol.

[11] Andajani-Sutjahjo S, Ball K, Warren N, Inglis V, Crawford D (2004). Perceived personal, social and environmental barriers to weight maintenance among young women: a community survey. Int J Behav Nutr Phys Act.

[12] Goode AD, Reeves MM, Eakin EG (2012). Telephone-delivered interventions for physical activity and dietary behavior change: an updated systematic review. Am J Prev Med.

[13] Eakin EG, Lawler SP, Vandelanotte C, Owen N (2007). Telephone interventions for physical activity and dietary behavior change: a systematic review. Am J Prev Med.

[14] Demark-Wahnefried W, Clipp EC, Morey MC, Pieper CF, Sloane R, Snyder DC, Cohen HJ (2006). Lifestyle intervention development study to improve physical function in older adults with cancer: outcomes from Project LEAD. J Clin Oncol.

[15] Goodwin PJ, Segal RJ, Vallis M, Ligibel JA, Pond GR, Robidoux A, Blackburn GL, Findlay B, Gralow JR, Mukherjee S, Levine M, Pritchard KI (2014). Randomized trial of a telephone-based weight loss intervention in postmenopausal women with breast cancer receiving letrozole: the LISA trial. J Clin Oncol.

[16] Grimmett C, Simon A, Lawson V, Wardle J (2015). Diet and physical activity intervention in colorectal cancer survivors: a feasibility study. Eur J Oncol Nurs.

[17] Harris MN, Swift DL, Myers VH, Earnest CP, Johannsen NM, Champagne CM, Parker BD, Levy E, Cash KC, Church TS (2013). Cancer survival through lifestyle change (CASTLE): a pilot study of weight loss. Int J Behav Med.

[18] Hawkes AL, Chambers SK, Pakenham KI, Patrao TA, Baade PD, Lynch BM, Aitken JF, Meng X, Courneya KS (2013). Effects of a telephone-delivered multiple health behavior change intervention (CanChange) on health and behavioral outcomes in survivors of colorectal cancer: a randomized controlled trial. J Clin Oncol.

[19] Morey MC, Snyder DC, Sloane R, Cohen HJ, Peterson B, Hartman TJ, Miller P, Mitchell DC, Demark-Wahnefried W (2009). Effects of home-based diet and exercise on functional outcomes among older, overweight long-term cancer survivors: RENEW: a randomized controlled trial. J Am Med Assoc.

[20] Rock CL, Byers TE, Colditz GA, Demark-Wahnefried W, Ganz PA, Wolin KY, Elias A, Krontiras H, Liu J, Naughton M, Pakiz B, Parker BA, Sedjo RL, Wyatt H, Exercise and Nutrition to Enhance Recovery and Good Health for You (ENERGY) Trial Group (2013). Reducing breast cancer recurrence with weight loss, a vanguard trial: the Exercise and Nutrition to Enhance Recovery and Good Health for You (ENERGY) Trial. Contemp Clin Trials.

[21] Goode AD, Lawler SP, Brakenridge CL, Reeves MM, Eakin EG (2015). Telephone, print, and web-based interventions for physical activity, diet, and weight control among cancer survivors: a systematic review. J Cancer Surviv.

[22] Paxton RJ, Nayak P, Taylor WC, Chang S, Courneya KS, Schover L, Hodges K, Jones LA (2014). African-American breast cancer survivors' preferences for various types of physical activity interventions: a Sisters Network Inc. web-based survey. J Cancer Surviv.

[23] Badr H, Chandra J, Paxton RJ, Ater JL, Urbauer D, Cruz CS, Demark-Wahnefried W (2013). Health-related quality of life, lifestyle behaviors, and intervention preferences of survivors of childhood cancer. J Cancer Surviv.

[24] Bantum EO, Albright CL, White KK, Berenberg JL, Layi G, Ritter PL, Laurent D, Plant K, Lorig K (2014). Surviving and thriving with cancer using a Web-based health behavior change intervention: randomized controlled trial. J Med Internet Res.

[25] De Cocker K, Charlier C, Van Hoof E, Pauwels E, Lechner L, Bourgois J, Spittaels H, Vandelanotte C, De Bourdeaudhuij I (2015). Development and usability of a computer-tailored pedometer-based physical activity advice for breast cancer survivors. Eur J Cancer Care (Engl).

[26] Forbes CC, Blanchard CM, Mummery WK, Courneya KS (2015). Feasibility and preliminary efficacy of an online intervention to increase physical activity in Nova Scotian cancer survivors: a randomized controlled trial. JMIR Cancer.

[27] Frensham LJ, Zarnowiecki DM, Parfitt G, King S, Dollman J (2014). The experiences of participants in an innovative online resource designed to increase regular walking among rural cancer survivors: a qualitative pilot feasibility study. Support Care Cancer.

[28] Hatchett A, Hallam JS, Ford MA (2013). Evaluation of a social cognitive theory-based email intervention designed to influence the physical activity of survivors of breast cancer. Psychooncology.

[29] Lee MK, Yun YH, Park H, Lee ES, Jung KH, Noh D (2014). A Web-based self-management exercise and diet intervention for breast cancer survivors: pilot randomized controlled trial. Int J Nurs Stud.

[30] Rabin C, Dunsiger S, Ness KK, Marcus BH (2011). Internet-based physical activity intervention targeting young adult cancer survivors. J Adolesc Young Adult Oncol.

[31] Valle CG, Tate DF, Mayer DK, Allicock M, Cai J (2013). A randomized trial of a Facebook-based physical activity intervention for young adult cancer survivors. J Cancer Surviv.

[32] Kanera IM, Bolman CA, Willems RA, Mesters I, Lechner L (2016). Lifestyle-related effects of the web-based Kanker Nazorg Wijzer (Cancer Aftercare Guide) intervention for cancer survivors: a randomized controlled trial. J Cancer Surviv.

[33] Kanera IM, Willems RA, Bolman CA, Mesters I, Verboon P, Lechner L (2017). Long-term effects of a web-based cancer aftercare intervention on moderate physical activity and vegetable consumption among early cancer survivors: a randomized controlled trial. Int J Behav Nutr Phys Act.

[34] Valle CG, Deal AM, Tate DF (2017). Preventing weight gain in African American breast cancer survivors using smart scales and activity trackers: a randomized controlled pilot study. J Cancer Surviv.

[35] Block G, Sternfeld B, Block CH, Block TJ, Norris J, Hopkins D, Quesenberry CP, Husson G, Clancy HA (2008). Development of Alive! (A Lifestyle Intervention Via Email), and its effect on health-related quality of life, presenteeism, and other behavioral outcomes: randomized controlled trial. J Med Internet Res.

[36] Sternfeld B, Block C, Quesenberry Jr CP, Block TJ, Husson G, Norris JC, Nelson M, Block G (2009). Improving diet and physical activity with ALIVE: a worksite randomized trial. Am J Prev Med.

[37] Thomas S, Reading J, Shephard RJ (1992). Revision of the physical activity readiness questionnaire (PAR-Q). Can J Sport Sci.

[38] Kushi LH, Doyle C, McCullough M, Rock CL, Demark-Wahnefried W, Bandera EV, Gapstur S, Patel AV, Andrews K, Gansler T, American Cancer Society 2010 Nutrition and Physical Activity Guidelines Advisory Committee (2012). American Cancer Society Guidelines on nutrition and physical activity for cancer prevention: reducing the risk of cancer with healthy food choices and physical activity. CA Cancer J Clin.

[39] Bandura A (2001). Social cognitive theory: an agentic perspective. Annu Rev Psychol.

[40] Bandura A (1989). Human agency in social cognitive theory. Am Psychol.

[41] Locke EA, Latham GP (2002). Building a practically useful theory of goal setting and task motivation. A 35-year odyssey. Am Psychol.

[42] Luca NR, Suggs LS (2013). Theory and model use in social marketing health interventions. J Health Commun.

[43] Prochaska JO, DiClemente CC (1992). Stages of change in the modification of problem behaviors. Prog Behav Modif.

[44] Ainsworth BE, Irwin ML, Addy CL, Whitt MC, Stolarczyk LM (1999). Moderate physical activity patterns of minority women: the Cross-Cultural Activity Participation Study. J Womens Health Gend Based Med.

[45] Centers for Disease Control and Prevention CDC.

[46] Food and Drug Administration, HHS (2003). Food labeling: trans fatty acids in nutrition labeling, nutrient content claims, and health claims. Final rule. Fed Regist.

[47] U.S. Department of Agriculture ARS ars.usda.

[48] Bowen DJ, Kreuter M, Spring B, Cofta-Woerpel L, Linnan L, Weiner D, Bakken S, Kaplan CP, Squiers L, Fabrizio C, Fernandez M (2009). How we design feasibility studies. Am J Prev Med.

[49] Wieland LS, Falzon L, Sciamanna CN, Trudeau KJ, Brodney S, Schwartz JE, Davidson KW (2012). Interactive computer-based interventions for weight loss or weight maintenance in overweight or obese people. Cochrane Database Syst Rev.

[50] Short CE, Rebar A, James EL, Duncan MJ, Courneya KS, Plotnikoff RC, Crutzen R, Vandelanotte C (2017). How do different delivery schedules of tailored web-based physical activity advice for breast cancer survivors influence intervention use and efficacy?. J Cancer Surviv.

[51] Short CE, Rebar A, James EL, Duncan MJ, Courneya KS, Plotnikoff RC, Crutzen R, Vandelanotte C (2017). How do different delivery schedules of tailored web-based physical activity advice for breast cancer survivors influence intervention use and efficacy?. J Cancer Surviv.

[52] Barnett SM, Ceci SJ (2002). When and where do we apply what we learn? A taxonomy for far transfer. Psychol Bull.

[53] Nigg CR, Lee H, Hubbard AE, Min-Sun K (2009). Gateway health behaviors in college students: investigating transfer and compensation effects. J Am Coll Health.

[54] Jayawardene WP, Torabi MR, Lohrmann DK (2016). Exercise in young adulthood with simultaneous and future changes in fruit and vegetable intake. J Am Coll Nutr.

[55] Fleig L, Lippke S, Pomp S, Schwarzer R (2011). Intervention effects of exercise self-regulation on physical exercise and eating fruits and vegetables: a longitudinal study in orthopedic and cardiac rehabilitation. Prev Med.

[56] Block G, Azar KM, Block TJ, Romanelli RJ, Carpenter H, Hopkins D, Palaniappan L, Block CH (2015). A fully automated diabetes prevention program, Alive-PD: program design and randomized controlled trial protocol. JMIR Res Protoc.

